# Addressing indigenous health workforce inequities: A literature review exploring 'best' practice for recruitment into tertiary health programmes

**DOI:** 10.1186/1475-9276-11-13

**Published:** 2012-03-15

**Authors:** Elana Curtis, Erena Wikaire, Kanewa Stokes, Papaarangi Reid

**Affiliations:** 1 Te Kupenga Hauora Māori (Department of Māori Health), Faculty of Medical and Health Sciences, University of Auckland, Private Bag 92019, Auckland 1142, New Zealand

**Keywords:** Māori, Indigenous, Under-represented Ethnic Minority, Recruitment, Health workforce development, Transitioning, Tertiary retention/completion

## Abstract

**Introduction:**

Addressing the underrepresentation of indigenous health professionals is recognised internationally as being integral to overcoming indigenous health inequities. This literature review aims to identify 'best practice' for recruitment of indigenous secondary school students into tertiary health programmes with particular relevance to recruitment of Māori within a New Zealand context.

**Methodology/methods:**

A Kaupapa Māori Research (KMR) methodological approach was utilised to review literature and categorise content via: country; population group; health profession ffocus; research methods; evidence of effectiveness; and discussion of barriers. Recruitment activities are described within five broad contexts associated with the recruitment pipeline: Early Exposure, Transitioning, Retention/Completion, Professional Workforce Development, and Across the total pipeline.

**Results:**

A total of 70 articles were included. There is a lack of published literature specific to Māori recruitment and a limited, but growing, body of literature focused on other indigenous and underrepresented minority populations.

The literature is primarily descriptive in nature with few articles providing evidence of effectiveness. However, the literature clearly frames recruitment activity as occurring across a pipeline that extends from secondary through to tertiary education contexts and in some instances vocational (post-graduate) training. Early exposure activities encourage students to achieve success in appropriate school subjects, address deficiencies in careers advice and offer tertiary enrichment opportunities. Support for students to transition into and within health professional programmes is required including bridging/foundation programmes, admission policies/quotas and institutional mission statements demonstrating a commitment to achieving equity. Retention/completion support includes academic and pastoral interventions and institutional changes to ensure safer environments for indigenous students. Overall, recruitment should reflect a comprehensive, integrated pipeline approach that includes secondary, tertiary, community and workforce stakeholders.

**Conclusions:**

Although the current literature is less able to identify 'best practice', six broad principles to achieve success for indigenous health workforce development include: 1) Framing initiatives within indigenous worldviews 2) Demonstrating a tangible institutional commitment to equity 3) Framing interventions to address barriers to indigenous health workforce development 4) Incorporating a comprehensive pipeline model 5) Increasing family and community engagement and 6) Incorporating quality data tracking and evaluation. Achieving equity in health workforce representation should remain both a political and ethical priority.

## Background

Indigenous^1 ^populations worldwide experience significant inequities in healthcare access, healthcare quality and ultimately health outcomes with persistent disparities observed in life expectancy, morbidity and mortality when compared to non-indigenous populations [1,2]. Addressing the underrepresentation of indigenous health professionals is recognised internationally as being an integral component of the overall response to overcoming indigenous health inequities [3]. However, indigenous students face significant barriers to participation and success in health education and understanding how to best achieve indigenous health workforce development remains a challenge.

This article explores the international literature for evidence of 'best-practice' for the recruitment of Māori (the indigenous population of New Zealand) or indigenous secondary students into health careers. Whether 'best practice', a contentious and potentially value-laden term, can actually be identified for Māori or indigenous students is an additional area of investigation for this study [4].

In New Zealand, Māori are under-represented within frontline health professional roles [5-7] (Table 1). The indigenous health workforce shortage in New Zealand is critical, with New Zealand having the largest proportion of overseas trained doctors internationally [8]. Evidence suggests that a lack of cultural concordance between patients and health professionals [9] may reduce patient satisfaction, access and adherence to treatments [10,11].

**Table 1:** New Zealand Māori and Pasifika health workforce representation

	Māori	Pasifika
Proportion of the New Zealand population	15%	7%

Proportion of Health professionals		
Doctors	2.6%	1.6%
Pharmacists	≤ 1.5%	0.4%
Nurses	7%	2.8%
Dentists	2.1%	0.6%

Therefore, under-representation of indigenous peoples within health professions reduces the potential of the health sector to provide a diverse, capable and culturally appropriate workforce that meets the needs of indigenous communities [12]. Similarly, the rights of indigenous peoples to equitable access to the opportunities of society, including the personal and community benefits of health workforce development and improving health outcomes may not be fully realised if such disparities continue [13-16].

There are multiple explanations for the shortage of indigenous health professionals reflecting a mixture of supply and demand issues associated with historical, political, demographic, cultural, academic and financial factors [17-23]. Initiatives aimed at increasing the recruitment of secondary school students into health careers are increasingly being funded by government agencies as a key mechanism to attract more indigenous students into what are often rigorous and demanding academic pathways [24,25]. At present, health recruitment initiatives in New Zealand tend to focus on making health careers 'attractive' to adolescents and are largely based within the secondary school sector. Recruitment programmes have only recently increased the focus on supporting indigenous secondary school students to achieve in the appropriate (e.g. science) subjects required for entry into health professional programmes.

**Figure 1:**
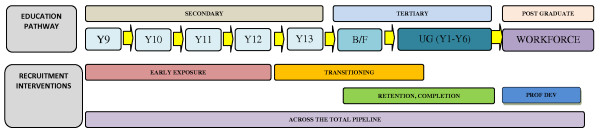
**Schemata of the recruitment pipeline - New Zealand context**.

A *pipeline *framework is commonly utilised to discuss health recruitment activity (Figure 1) [3,26].

The Sullivan Commission report [27] notes that *'a pipeline from primary to secondary to postsecondary education, and finally to professional training, channels the flow of a diverse and talented stream of individuals into the nation's health care workforce' *(p. 72). However, exactly what specific components are most effective and efficient, where they should be provided and how recruitment should be occurring within this pipeline remains unclear [13]. Furthermore, lessons can be learnt from other countries attempting similar (or different) approaches to the production of indigenous health professionals. This literature review considers broad recruitment contexts that may inform the health sector on how best to recruit indigenous secondary school students into health careers. Framed from a New Zealand context with a particular focus on Māori student recruitment, it is expected that the findings will have relevance internationally for other institutions hoping to address similar issues.

## Research design

### Aim

The aim of this literature review is to identify national and international evidence of best practice for recruitment of Māori or indigenous secondary school students into health related tertiary programmes.

### Methodology

A Kaupapa Māori Research (KMR) [28] methodological approach was utilised. KMR is based on a number of key principles [29]. In this instance it aims to provide: a critique of literature from a Māori paradigm that takes into account the structural and power imbalances within society that perpetuate inequities; an explicit challenge to or rejection of 'victim blame' and 'cultural deficit' analyses [30]; a commitment to ensuring that the research outputs will have positive benefits for Māori communities; a commitment to Māori, research leadership and workforce development. Such an approach involves questioning conclusions that require students to change their behaviour rather than the recruitment programme, inclusion of non-published but highly relevant Māori literature, acknowledging the relevance of other indigenous and under-represented minority population literature whilst maintaining a focus on Māori as indigenous peoples in the New Zealand context, rejection of findings that suggest the culture of Māori students is to blame for their educational failures and ensuring that any recommendations made from the review aim to facilitate Māori student success.

### Data sources

Literature were initially identified via searches within three sources: (1) Medline (Ovid) database, (2) ERIC database and (3) grey literature (unpublished documents or reports highly relevant to the aim).

Initial searches specific to Māori or other indigenous populations (i.e. Aboriginal, Torres Strait Islander, Native American, Inuit, Metis or First Nation peoples) produced very few results. The search was therefore widened to include Pacific (i.e. the heterogeneous composite of Pacific ethnic minority groups living in New Zealand e.g. Samoan, Tongan, Nuiean, Cook Island), however this also provided only a small number of articles. Given this context, the research team decided that the literature search would extend to under-represented minority groups or URM. ^2 ^Data on URM students have been included as their situations, statistics and challenges often mirror those of indigenous students. Furthermore, issues associated with power, privilege and agency within society are hypothesised to be similar for indigenous and URM groups and therefore exploring the literature for these groups collectively was thought to be appropriate given the small amount of data currently available. The term indigenous/URM is used when discussing the actual literature review findings with specific reference to Māori where appropriate.

Key search words included: 'Māori', 'Indigenous', 'Aboriginal', 'Minority', 'Ethnic', 'Race', Disadvantaged', 'Underrepresented', 'Recruit*', 'Secondary', 'Tertiary', 'Undergraduate', 'School', 'Student', 'Education', 'Programme', 'Health', 'Science', 'Careers', 'Selection', 'Transition', 'Retention', 'Admission', 'Best Practice', 'Intervention'. A total of 216 articles from Medline and 202 articles from ERIC were initially identified using various combinations of these key words. All abstracts were reviewed and articles were excluded using the following criteria: published before 1985; not specifically relevant to the stated aim; not in English language; could not be obtained in hard copy. Application of the above criteria resulted in 172 Medline and 195 ERIC articles being excluded. A total of 70 articles sourced from Medline (44), ERIC (7) and Grey Literature (19) remained for inclusion in this literature review [5,6,12,14,17,18,20-22,25,26,31-90].

### Analysis

All data (hereon referred to as 'articles') were reviewed and article content was categorised via the following variables: country; population group; health profession focus; research methods; evidence of effectiveness; and discussion of barriers (Table 2). Recruitment activities were categorised into five broad contexts associated with the recruitment pipeline (Table 3). The potential overlap of activities between these broad contexts is acknowledged, however, the following definitions have guided this literature review: (1) early exposure (i.e. activities targeted towards secondary school students that aim to expose students to health careers and academic pathways), (2) transitioning (i.e. activities that assist secondary school students to enter tertiary study programmes), (3) retention/completion (i.e. activities that aim to support success in tertiary health professional programmes), (4) professional workforce development (i.e. activities that aim to develop the existing health workforce including continuous professional development, specialisation or career development) and (5) across the total pipeline (i.e. activities that occur in more than one aspect of the pipeline).

**Table 2:** Literature focus

Description of available literature	No. of articles
**Country**	
United States of America	37
Australia	15
New Zealand	13
United Kingdom	3
Canada	2

**Population group**	
Underrepresented minorities*	37
Indigenous^®^	24
Māori˜	12
Pacific^	11

**Health profession focus**	
Medicine	40
Nursing/Midwifery	31
Dentistry	24
Allied health professions§	20
Pharmacy	11

**Research methods**	
Descriptive	55
Student data analysis	34
Surveys	24
Interviews	14
Focus Groups	11
Workshops/seminars	10

**Evidence of effectiveness**	
New evidence of effectiveness	42
Process of recruitment	35
Promising workforce outcomes	26
Workforce achievement	10

**Discussion of barriers**	42

* Ethnic, non-indigenous, non-dominant groups who make up a small proportion of the population representing a mixture of African-American, Hispanic and/or Asian/US Pacific.

^® ^The original inhabitants of a country representing a mixture of Aboriginal, Torres Strait Islander, Native American, Inuit, Metis or First Nation peoples.

^~ ^Indigenous population of New Zealand

^ The heterogeneous composite of Pacific ethnic minority groups living in New Zealand (e.g. Samoan, Tongan, Cook Island Māori)

§ A mixture of Physiotherapy, Occupational Therapy, Mental Health, Osteopathic Medicine, Health Sciences

**Table 3:** Literature content

Recruitment activities	No. of articles
**Early exposure**	
School visits	41
Additional academic preparation	40
Secondary enrichment programme	38
Community involvement	35
Careers advisors	36
Parental involvement	34
Advertising/marketing	27
Secondary financial support	7

**Transitioning**	
Admission/quota policies	36
Tertiary enrichment programmes	30
Pre-matriculation/application support	28
Bridging/foundation programmes	25

**Tertiary retention/completion**	
Support programmes	49
Staff/faculty	48
Community involvement	47
Tertiary financial support	39
Curriculum development	35
Tertiary mission statement/vision	17
Accommodation support	6
First year expansion	4

**Professional workforce development**	12

**Across the total pipeline**	
Role models	53
Mentoring	50
Health work experience	30
Evaluation/tracking	25
Research	24
Spiritual/cultural values	19

## Results

### Literature focus

Table 4 provides a synopsis of the specific recruitment activities occurring within these five contexts. A summary of the content associated with each article based on the variables and contexts identified above is provided in Table 5.

**Table 4:** Synopsis describing recruitment activities within five broad contexts

Early exposure	Activities targeted towards secondary school students that aim to expose students to health careers and academic pathways
School visits	Visiting schools for recruiting purposes

Parental involvement	Including parents/families in recruitment activities

Career advisors	Including careers advisors in recruitment activities

Financial support	Providing financial assistance to secondary school students

Additional academic preparation	Providing additional academic support to secondary school students (e.g. science camps, tutoring)

Secondary enrichment programmes	Providing extracurricular health career activities onsite at university

Advertising/marketing	Including marketing/advertising to secondary school students

**Transitioning **	Activities that assist secondary school students to enter tertiary study programmes

Tertiary enrichment programmes	Providing health career activities onsite to expose students to tertiary environment and health career programmes

Bridging/Foundation programmes	Programmes that bridge the transition from secondary school into tertiary study. Usually provide extra academic support to fill educational gaps.

Pre-matriculation/Application support	Programmes that support students to enter into health professional training. but may also include shorter targeted programmes for entry into medicine/dentistry etc.

Quota/Admissions policy	Specific quota for admission and/or admission policy to increase numbers of indigenous students

**Tertiary**	Activities that aim to support success in tertiary health professional programmes

Retention/support	Tertiary retention and support interventions for enrolled students (e.g. MAPAS).

Tertiary financial support	Financial assistance for tertiary students (e.g. scholarships, resources, stipends)

First year expansion	Option to complete first year of health study over an extended timeframe (e.g. two years).

Curriculum	Curricula that are inclusive of indigenous/URM perspectives, content etc.

Faculty staff	Increased numbers of indigenous/URM faculty staff members (who act as role models, support for students and recruitment programmes).

Accommodation support	For living arrangements whilst studying health programmes or during placements/specific learning interventions

**Professional workforce development**	Activities that aim to develop the existing health workforce including continuous professional development, specialisation or career development

**Across the total pipeline **	Activities that occur in more than one aspect of the pipeline

Spirituality/Cultural values	Spirituality/cultural values/indigenous perspectives are acknowledged and incorporated into health professional training programmes.

Tertiary Mission Statement	A tertiary institution mission/vision statement demonstrating their commitment to Indigenous workforce development

Community involvement	Involve community groups/individuals or organisations in recruitment activities

Mentoring	Mentoring for students within their programmes (formal and informal)

Work exposure	Health sector work experience based recruitment activities for students

Role models	Access to role models (i.e. other Indigenous/URM health practitioners or faculty members)

Research	Conducting research as part of the recruitment programme.

Evaluation/tracking	Evaluation/tracking as part of the recruitment programme.

**Table 5:** Summary of individual article content based on variables and context identified

Article Descriptor	Population			Professional Focus						Methods						Pipeline Detail				Evidence of Effectiveness			
**Article Name/Year**	**Māori**	**Pacific**	**Indigenous**	**URM**	**Medicine**	**Nursing**	**Dentistry**	**Pharmacy**	**Allied Health**	**Descriptive**	**Data analysis**	**Focus Group**	**Survey**	**Interviews**	**Workshop**	**Early Exposure**	**Transitioning**	**Tertiary**	**Across Pipeline**	**New**	**Process**	**Promising**	**Achievement**

Acosta (2006)			•		•					•	•					•	•	•	•	•		•	•

AIDA (2005)			•		•					•			•	•	•	•	•	•	•				

AIDA (2010)			•		•					•						•	•	•	•				

Andersen (2009)				•			•			•	•		•	•		•	•	•	•	•		•	

Andersen (2007a)				•			•			•			•					•		•	•		

Anderson (2007b)			•		•					•						•		•	•				

Beacham (2009)				•		•				•	•					•	•	•	•				

Bediako (1996)				•	•					•	•					•				•		•	

Brunson (2010)				•			•			•	•					•	•	•	•	•		•	

Campbell (2008)				•		•				•					•	•		•	•	•	•	•	

Chesters (2009)			•		•					•	•		•			•				•			

Collins (1997)	•	•			•		•			•	•	•	•			•	•	•		•		•	•

Cooney (2006)				•	•	•	•	•	•				•			•	•		•	•	•		

Cooper (2003)				•	•					•	•					•	•	•					

D'Antoine (2006)			•		•		•		•	•	•					•	•		•	•	•	•	•

DeLapp (2008)			•			•				•						•		•	•	•	•	•	•

Drysdale (2006)			•		•					•	•	•	•			•	•	•	•	•	•		

Erwin (2004)				•	•	•				•	•		•			•	•	•	•	•	•		

Farrington (1999)			•			•			•					•		•	•	•	•	•	•	•	•

Fields (2001)				•		•			•							•	•	•	•				

Fitzjohn (2003)			•		•			•									•	•		•	•		

Fletcher (2003)				•		•		•								•	•	•	•				

Friedland (1997)			•	•				•								•	•		•				

Gabard (2007)			•	•	•	•	•	•	•	•						•	•	•	•				

Gordon (2010)			•		•				•							•	•	•	•	•	•	•	•

Gravely (2004)			•			•					•						•	•	•	•	•	•	

Greenhalgh (2004)			•	•												•			•	•	•	•	

Greenhalgh (2006)		•	•	•			•	•	•		•					•	•	•	•				

Haskins (2006)			•	•			•	•	•		•					•		•	•	•	•		

Hollow (2006a)		•	•	•			•	•	•		•					•	•	•	•	•	•	•	•

Hollow (2006b)		•		•							•					•	•	•	•	•	•		

INEWG (2002)		•	•	•				•	•	•	•					•	•	•	•				

Kelly (2009)		•	•	•	•	•	•	•		•						•	•	•	•				

Kivell (2008)		•	•	•	•	•	•																

Lawson (2007)				•	•	•	•	•	•							•	•	•	•				

Lewis (1996)																•	•	•	•	•	•	•	•

Lopez (2003)																							

Mackean (2007)							•		•						•	•							

McClintock (2008)																							

Nat. ATSI Hlth											•									•			

Council (2008)																							

Nikora (2002)																							

Nnedu (2009)				•		•	•																

Noone (2008)			•	•	•	•											•	•	•	•	•	•	

Noone (2007)				•													•	•	•	•	•	•	

Omeri (1999)											•												

Poole (2009)																							

Price (2008)			•			•			•	•		•		•	•	•	•	•	•	•	•		

Ratima (2007)	•	•		•				•	•						•	•	•	•					

Royal Soc. Of NZ (2005)	•	•		•	•	•	•		•	•				•	•	•	•	•					

Rumala (2007)		•	•	•					•	•	•	•	•		•	•	•	•	•	•	•		

Sapoaga (2011)		•						•		•						•	•	•	•	•	•		

Sewell (2008)			•			•			•	•					•	•	•	•					

Shields (1991)			•						•	•					•		•	•	•	•	•		

Soto-Greene (1999)			•	•			•			•	•				•	•	•	•	•	•	•		

Spencer (2005)		•		•						•	•		•		•	•	•	•					

Stewart (2004)			•				•						•		•			•	•	•			

Te Rau Matatini (2009)	•					•							•	•	•	•	•	•	•	•			

Sulliv. Commiss (2004)		•	•	•		•	•			•	•			•	•	•	•	•					

Thompson (1993)			•	•		•	•	•	•				•		•			•	•	•			

Thompson (1999)			•						•	•	•					•	•	•	•	•	•	•	

Turale (2006)		•				•				•			•	•	•		•	•					

Usher (2005a)		•				•				•	•		•				•	•	•	•	•		

Usher (2005b)		•				•				•	•				•	•	•	•					

Wadenya (2008)			•				•					•	•	•	•		•	•	•	•	•		

Waetford (2007a)	•	•		•		•	•	•	•	•	•				•	•	•	•	•		•		

Waetford (2007b)	•			•		•	•	•	•	•					•		•	•					

Werry Centre (2008)	•	•							•	•					•			•					

Wiggs (2000)			•	•		•	•	•	•	•	•	•	•		•	•	•	•	•	•			

Winkleby (2007)			•	•		•	•	•	•	•	•				•			•	•	•	•	•	

Zuzelo (2005)			•			•				•					•		•	•					

The majority of articles are sourced from the United States of America (USA) (37/70), followed by Australia (15/70) and New Zealand (13/70) with only a small number of articles obtained from the United Kingdom (3/70) and Canada (2/70). Most articles referred to *Under Represented Minority *(37) population groups with 24 articles specific to *Indigenous *population groups. A smaller number of articles were identified if they specifically referred to *Māori *(12) and *Pacific *(11) population groups. Health profession areas of focus include *medicine *(40), followed by *nursing/midwifery *(31), *dentistry *(24), *allied health professions *(20) and *pharmacy (*11).

The majority of articles are *descriptive *in nature (55). Thirty-four articles provide some evidence and/or analysis of *tertiary student data*. Twenty-four articles involved *surveys *of students, families, programme and/or university staff with a smaller number of sources presenting findings from *interviews *(14), *focus groups *(11) and *workshop/seminars *(10).

Of the 70 articles, 42 sources provide new *evidence of effectiveness *regarding the recruitment activities described. Of these, 35 sources discuss the appropriateness of the process of recruitment, 26 sources present evidence of promising workforce outcomes (e.g. matriculation/enrolment into health programmes) and 10 present evidence of workforce achievement (e.g. completion/graduation from health programmes).

### Literature content

Nearly three quarters of the articles present their findings within the context of a pipeline that integrates recruitment across both the *secondary and tertiary education *sectors (51/70) compared to articles that have a specific focus on either *secondary education *(7/70) or *tertiary education *(12/70). Overall, there is a similar amount of evidence that refers to recruitment activities within *early exposure *(59/70), *transitioning *(47/70), *retention/completion *(60/70) and those activities that occur *across the total pipeline *(66/70). A small number of articles discuss the importance of *professional workforce development *(12/70) within the recruitment pipeline; however, these articles are not the key focus of this literature review, and therefore a more extensive literature review focused on professional workforce development is required to examine this aspect of the pipeline further.

#### Early exposure

Early exposure interventions focused on *school visits*, provision of *additional academic preparation *and targeted *secondary enrichment programmes *where students are provided with opportunities to visit tertiary institutions and health settings. Improving advice from *careers advisors*, including *parental involvement *and *advertising/marketing *to support career decision making were highlighted. A small number of USA articles referred to the provision of *secondary financial support *to students whilst studying at secondary school.

#### Transitioning

*Admission/quota policies *targeted towards indigenous and URM students and the offering of *tertiary enrichment programmes *to undergraduate students seeking post-graduate entry into health training were well described. Student *pre-matriculation/application support *and the provision of *bridging/foundation programmes *to address academic prerequisite gaps (particularly within the science subjects) were other key transitioning activities.

#### Retention/completion

Tertiary retention and completion activities included the provision of specific *support programmes *and the need to develop *staff/faculty *with a particular focus on indigenous and URM academic leadership. Retention activities referred to the provision of *tertiary financial support *and a commitment to indigenous/URM *curriculum development*. The provision of *accommodation support *and the options of *first year expansion *particularly within medical programmes were discussed in a smaller number of articles.

#### Across the pipeline

A number of activities occur across the total pipeline and are used within secondary, transitioning and tertiary contexts including the use of indigenous and URM *role models, mentoring *and ensuring *community involvement *in recruitment activities. Facilitating access for secondary and tertiary students to obtain *health work experience *is encouraged. Some articles specifically noted the need to conduct ongoing *evaluation/tracking, research*, inclusion of *spiritual/cultural values *within the recruitment process and the presence of a *tertiary mission statement/vision*.

## Discussion

This study reviewed 70 articles to explore national and international best practice for recruitment of Māori or indigenous/URM secondary school students into health related tertiary programmes. The following discussion explores key issues relevant to the recruitment pipeline in total and within the specific contexts of early exposure, transitioning, retention/completion.

### Early exposure

The literature identifies a number of barriers for indigenous/URM secondary school students wishing to pursue health careers. These barriers reflect education, information, aspiration and access issues requiring the development of appropriate interventions within the early exposure context.

For example, within the New Zealand context, inequities in academic achievement rates between Māori and non-Māori secondary school students, particularly within science subjects, are a major barrier underlying the need for early exposure. In 2007 nearly two out of five Māori aged ≥ 15 years had no formal qualification and participation in a Year 13^3 ^science subject was only 23% for Māori secondary school students compared to 41% for non-Māori [91]. In 2009, only 29% Māori versus 54% non-Māori students received university entrance at the completion of their final year of secondary school study [92,93]. Similar academic achievement inequities exist internationally and therefore interventions that encourage indigenous students to choose the appropriate prerequisite subjects at secondary school such as sciences (i.e. Chemistry, Biology, and Physics) and other relevant subjects are needed (e.g. English and Mathematics) [19,71,83,84]. Once students have chosen the right subjects, appropriate support should be provided to ensure indigenous students attain the necessary credits for entry into health programmes [27,83]. Early exposure must therefore ensure that secondary school students have access to the necessary academic building blocks for movement into health professional programmes.

The literature also discusses the importance of actively including parents, families and indigenous/URM communities in early exposure activities due to their influence on student career choices [13,19,37,60,78,83,90]. This is particularly important as indigenous/URM students are often diverted away from health careers via careers advisors and teachers [18,19]. For example, Chesters et al. [35] found that only 18% (26/144) of secondary school advisors or guidance counsellors were able to demonstrate the knowledge required to effectively advise or support indigenous students into health careers. Similar findings are described elsewhere [18,19,27,44,60,64,74,78,83] with Hollow et al. [44] noting *"when counsellors or teachers "track" minority [or indigenous] students into less rigorous academic courses - intentionally or not - they limit student's academic achievement and inhibit their career aspirations"(p. 4)*.

Addressing the deficit or failure discourse of career advisors via professional development [35] and, more importantly, supporting the use of indigenous/URM staff, role models or mentors to provide careers advice is recommended [21,44,83,94].

Other enrichment activities such as visits to tertiary institutions that expose indigenous/URM students to campus environments early [17,70,71,95] are recommended. These enrichment activities are important as they introduce students to indigenous/URM role models they may not have had access to previously [47,56,67,82], they foster trust between tertiary providers and families/communities [47,56] and enhance student confidence and motivation to apply for health programmes [42].

### Transitioning

Indigenous/URM students face significant barriers when transitioning into tertiary programmes. While many students entering university have to leave their families, communities and support networks; indigenous/URM students have the additional pressure of entering into what is generally a non-indigenous/URM, foreign and unfriendly tertiary environment [18,51,96]. Madjar et al. [96] discuss the large size of first year degree lectures as contributing to student's feelings of isolation [96], with indigenous/URM students often feeling *'culturally alienated' *(p. 1049) within the tertiary environment [97]. Indigenous/URM specific orientations may therefore create a more welcoming and indigenous/URM appropriate introduction to tertiary settings [17,41,71].

Indigenous/URM students experiencing academic barriers to programme entry (often complicated with additional pastoral and socio-economic issues) [13,95] may be best supported by a bridging/foundation programme. To achieve this comprehensive support, bridging/foundation programmes differ according to their duration (from 4-6 weeks to an academic year), content focus (individualised to a specific student versus generic programme provision) and cohort size (from 6 students to over 100 students) [95,98] and are often designed for their local contexts. Many utilise indigenous/URM curriculum content (e.g. Aboriginal health topics) [13,61,74,96], specifically support the acquisition of study skills required for tertiary study [71,86], whilst also ensuring that students are given access to indigenous/URM role models for motivation to continue along the pathway towards health careers [13,48]. Bridging/foundation programmes therefore have the potential to provide comprehensive transitioning support to address gaps in educational achievement whilst also ensuring that students are set up for success from a pastoral perspective.

Tertiary institutions should demonstrate a tangible commitment to equity initiatives via the provision of well defined, accessible and targeted admission policies or quotas for indigenous students. Support to navigate the often complex university application processes should also be provided [81]. These activities not only create direct pathways for indigenous/URM student entry, they also promote the institution as valuing cultural diversity which has been identified as being attractive for indigenous/URM students considering health as a career [17,31].

It is acknowledged that transitioning in a broader context occurs at multiple sites along the pipeline whenever students move between environments within the education sector (e.g. secondary to tertiary, undergraduate students into graduate health study). Therefore institutions may need to provide multiple forms of transitioning support beyond the definition outlined within this literature review [27,96].

### Retention/completion

Inequities in academic achievement for indigenous/URM students persist into the tertiary context. New Zealand statistics for example, show that national participation rates for Māori aged 18-19 in degree level study is less than half the rate for all students, Māori first year attrition rates in bachelor degree level programmes are 28.8% versus 18.2% for non-Māori [99,100] and Māori rates of participation in health professional degree level programmes remain low [101]. This data supports the need for successful recruitment programmes to extend beyond getting students *into *programmes and provide indigenous students with comprehensive retention and completion support within the tertiary context [17,18,73].

Similar to other educational settings [102], support for indigenous/URM students within health professional programmes should include academic interventions such as additional tutorials [69,84], study support [84] and formation of study groups [21,64]. Pastoral interventions should also be provided to facilitate student access to financial assistance (a pivotal support requirement) [54,69,86,90], referral to counseling as required [51,80,84] and provision of ongoing career advice [51,69]. Tertiary institutions need to provide the resources to support retention activities including the provision of culturally safe spaces for indigenous students [51,69]. Having access to an indigenous/URM specific space encourages peer support, provides a safe haven from any racism experienced from their non-indigenous/URM peers or institution and allows students to operate within culturally appropriate contexts (e.g. physical study spaces that include shared eating opportunities) [13,18,51,83].

However, encouraging indigenous/URM students to become more resilient to unsafe environments and negative experiences within their health professional training is an inadequate response [18]. Tertiary institutions can address these issues and target student development via curriculum reform that includes both indigenous/URM and cultural safety content [17,83]. The provision of professional development for staff to understand and address these issues is also required [17,51,54,71] alongside the development of indigenous/URM academic and general staff positions [17,18,56] and the promotion of indigenous/URM academics to positions of tertiary leadership [56]. Whilst many of these interventions are not new to those delivering tertiary support, the framing of these interventions within a secondary school recruitment framework reflects the broader pipeline approach.

### The pipeline

Debate exists around the definition and scope of 'receruitment' with some commentators suggesting that recruitment activities cease once students enter a tertiary institution [103]. However, the literature clearly frames recruitment activity as occurring across a pipeline that extends from secondary through to tertiary education contexts and in some instances vocational (post-graduate) training [13,21,25,27,74,82,85,104].

Given this context, tertiary institutions have a responsibility (and arguably a requirement) to provide both secondary school recruitment *and *tertiary retention initiatives that target indigenous/URM students. This reflects ethical concerns that may arise if institutions actively recruit students into tertiary environments that fail to ensure indigenous student success. Similarly, recruitment programmes have a responsibility to ensure that they are knowledgeable of, and well integrated with, the tertiary sector for successful workforce development. There is little point in making health attractive to students if they have no realistic chance or pathway for entry into what are often highly competitive programmes.

Recruitment should be framed from a comprehensive and integrated pipeline perspective that has the ability to include secondary, tertiary, community and workforce stakeholders. Active inclusion of indigenous/URM communities throughout the recruitment pipeline is encouraged [19,32,68], with some articles highlighting the need to better incorporate indigenous spiritual and cultural practices within secondary and tertiary contexts [19,61]. Using a specific mission statement or vision to demonstrate a commitment to equity initiatives may assist institutions to maintain support for an integrated, indigenous/URM-appropriate, recruitment approach [13,51,71,85,90].

A number of common recruitment activities operating across the pipeline are described in the literature. For example, both secondary and tertiary students are noted to benefit from exposure to indigenous/URM role models, mentors and work experience in health settings [17,27,35,69]. Unfortunately, clear and detailed descriptions of these specific activities are rarely provided and therefore assume a common understanding. 'Mentoring' for example, can be delivered both formally and informally, and may involve student peers, staff or health professionals [105]. Understanding exactly what programmes *mean *by mentoring is important as different models will have different protocol and resourcing implications.

Given the limited evidence of effectiveness obtained from this literature review, recruitment programmes and tertiary institutions need to improve data tracking and research potential to better inform recruitment initiatives [69]. New Zealand in particular needs to increase its contribution to the published literature in order to better present and examine the numerous recruitment strategies targeted at Māori students.

## Strengths and limitations

This study highlights the lack of published literature specific to Māori recruitment and the limited, but growing, body of literature focussed on other indigenous and under-represented minority populations. The majority of articles refer to URM groups, are based in the USA and tend to focus on medicine, nursing/midwifery and dentistry. Generalisation of these findings to other settings such as indigenous students, the New Zealand context and broader health professions may be problematic. Nevertheless, it remains important to consider these programmes as models of what may (or may not) be achieved when working with populations facing similar power, privilege and agency within society and barriers to health care professional training with the proviso that adaptation to local contexts will be required.

The literature is primarily descriptive in nature with few articles providing evidence of effectiveness [17,18,26,69,73,95]. Therefore, it is arguable that the current literature cannot fully describe 'best practice' [4]. Given this context, broad principles to inform and enhance the potential of recruitment programmes to achieve success for indigenous health workforce may be more preferable than the identification of "best practice" per se. The challenge remains to more fully understand the various components, their contributions and interactions along the recruitment pipeline, including a broader discussion of what constitutes good practice from an indigenous perspective.

It is acknowledged, that indigenous recruitment programmes are relatively new and may be less able to conduct and publish research whilst in developmental phases, particularly if there is a lack of institutional support for formal evaluation and tracking of outputs [81]. These issues need to be addressed if we are to fully understand how to best recruit and graduate indigenous students into health careers. A small number of articles described recruitment activities that targeted students within primary school settings however this requires further investigation that was outside the scope of this study.

Despite these concerns, this literature review has a number of strengths including the use of Kaupapa Māori methodology that critiques the literature from an indigenous and specifically Māori perspective; the use of a systematic review process to consider international literature as a body of evidence rather than reviewing articles in isolation; and the timeliness of reviewing the available evidence given the increasing political and health sector support for funding of indigenous health workforce development initiatives [106,107].

## Conclusions

Addressing the underrepresentation of indigenous health professionals is recognised internationally as being integral to overcoming indigenous health inequities [3,27,81,107]. The literature supports the provision of indigenous specific recruitment programmes as a key initiative for indigenous health workforce development. Although the current literature is less able to identify 'best practice', six broad principles can be drawn from the literature to inform and enhance the potential of recruitment programmes to achieve success for indigenous health workforce development:

1. Frame recruitment initiatives within an indigenous worldview that takes into account indigenous rights, realities, values, priorities and processes.

2. Demonstrate a tangible institutional commitment to achieving indigenous health workforce equity via the development (and proactive support of) a mission statement/vision and appropriate policies and processes.

3. Identify the barriers to indigenous health workforce development and use these to frame recruitment initiatives within your local context.

4. Conceptualise and incorporate recruitment activity within a comprehensive and integrated pipeline model that operates across secondary and tertiary education sectors via the provision of early exposure, transitioning, retention/completion and post-graduation activities.

5. Increase engagement with parents, families and indigenous communities (including tribal groups) within all recruitment activities but particularly early exposure.

6. Incorporate high quality data collection, analysis and evaluation of recruitment activities within programmes with the publication of results where possible.

International institutions should apply these principles to indigenous health workforce development initiatives in a way that is meaningful, appropriate and effective within their own indigenous context. Successful indigenous health workforce recruitment has a key role to play in overcoming health inequities for indigenous peoples. Achieving equity in health workforce representation must therefore remain both a political and ethical priority.

## Abbreviations

ERIC: Education Resources Information Center; KMR: Kaupapa Māori Research; URM: Under-represented minorities; USA: United States of America.

## Endnotes

^1^There are multiple descriptors for the term indigenous. Common themes include: those cultures whose world views place special significance on the idea of the unification of the humans with the natural world (Royal, C. (2003). Indigenous worldviews; A comparative study. Otaki, New Zealand: Te Wānanga-o-Raukawa) and descendants of those who inhabited a country or a geographical region at the time when people of different cultures or ethnic origins arrived (United Nations. (2004). The Concept of Indigenous Peoples: Background paper prepared by the Secretariat of the Permanent Forum on Indigenous Issues. Workshop on data collection and disaggregation for Indigenous Peoples (New York, 19-21 January 2004), United Nations, Department of Economic and Social Affairs.).

^2^Medical definitions for the term URM are changing over time. One definition includes "those racial and ethnic populations that are underrepresented in the medical profession relative to their numbers in the general population" (Association of American Medical Colleges. (2003). Underepresented in Medicine Definition. Retrieved from https://www.aamc.org/initiatives/urm). For the purposes of this article, URM refers to a mixture of African-American, Hispanic and/or Asian/US Pacific. Indigenous populations that may have been included within URM articles have been dually identified as indigenous for this analysis.

^3^Year 13 is the final year of secondary schooling in Aotearoa New Zealand.

## Competing interests

The authors declare that they have no competing interests.

## Authors' contributions

EC developed and led the concept development and study design, carried out the literature review and analysis and drafted the manuscript. EW completed the literature content analysis, and contributed to the literature search, drafting and revising the manuscript. KS completed the literature search and initial inclusion/exclusion of articles, participated in the study design and analysis and contributed to drafting and revising the manuscript. Associate Professor Papaarangi Reid participated in the study design and methodological approach. PR also contributed to the drafting and revision of the manuscript for important intellectual content. All authors read and approved the final manuscript.

## Authors' information

Dr Elana Curtis (Te Arawa) is a specialist in public health medicine who has experience in research and policy concerned with eliminating ethnic and indigenous inequalities in health. Elana is currently employed as a Senior Lecturer and is Director of the *Vision 20:20 *initiative at Te Kupenga Hauora Māori, The University of Auckland. Ongoing research includes ethnic disparities in cardiovascular disease, use of Kaupapa Māori Research methodology and indigenous health workforce development.

Erena Wikaire (Ngāti Hine) is a Māori Physiotherapist who has experience in research concerned with Māori and Indigenous health workforce development, cultural competence, and psycho-oncology in Māori and Indigenous populations. Erena is currently completing a Masters in Public Health whilst working as a Researcher at Te Kupenga Hauora Māori, University of Auckland. Ongoing research interests include Māori health workforce development and addressing ethnic inequalities in health.

Kanewa Stokes (Ngāti Porou/Te Whānau ā Apanui) is the Development Manager for the Whakapiki Ake Project for recruitment of Māori into health careers within Te Kupenga Hauora Māori, The University of Auckland. Kanewa holds a Masters degree in social policy and has worked in research and evaluation across the Māori and indigenous education, health and social development sectors.

Associate Professor Papaarangi Reid (Te Rarawa) is Tumuaki and Head of Department of Māori Health at the Faculty of Medical and Health Sciences, University of Auckland, New Zealand.She holds science and medical degrees from the Universities of Otago and Auckland and is a specialist in public health medicine. Her research interests include analysing disparities between indigenous and non-indigenous citizens as a means of monitoring government commitment to indigenous rights.
